# Treg–microglia crosstalk in Alzheimer’s disease: stage-dependent dynamics, molecular mechanisms, and translational challenge

**DOI:** 10.3389/fnagi.2026.1838306

**Published:** 2026-05-25

**Authors:** Hua Fu, Jing Yang, Miao Zhang

**Affiliations:** 1 The Second Clinical Medical College, Heilongjiang University of Chinese Medicine, Harbin, Heilongjiang, China; 2 The Second Affiliated Hospital of Heilongjiang University of Chinese Medicine, Harbin, Heilongjiang, China

**Keywords:** Alzheimer’s disease, immune regulation, microglia, neuroinflammation, regulatory T cells

## Abstract

Alzheimer’s disease (AD) is characterized by amyloid-beta (Aβ) deposition, tau hyperphosphorylation, and chronic neuroinflammation. Emerging evidence from preclinical models suggests that aberrant immune crosstalk between regulatory T cells (Tregs) and microglia may contribute to disease progression, though its precise role in human AD remains to be fully elucidated. In rodent models, Tregs have been shown to cross the blood-brain barrier and, through cell-contact-dependent mechanisms and secretion of pro-resolutive factors such as transforming growth factor-β, appear to promote microglial transitions toward pro-resolutive states and facilitate Aβ phagocytosis. However, these mechanisms have been predominantly demonstrated in transgenic mouse strains with early-onset amyloid pathology, and their relevance to the slow, aging-associated progression of human sporadic AD requires cautious interpretation. In AD animal models, reductions in Treg numbers and suppressive function coincide with microglial dysregulation, with the interaction between these cell types shifting from homeostatic to pro-inflammatory states as pathology advances. It is critical to note that while such findings suggest a potential regulatory axis, they derive largely from simplified animal systems that do not fully recapitulate human immune aging, genetic heterogeneity, or decades-long disease kinetics. Moreover, therapeutic strategies targeting this crosstalk that show efficacy in mice have yielded inconsistent results in early human trials, highlighting significant translational gaps. This review critically assesses the current preclinical evidence, emphasizing that findings from rodent models should be interpreted as hypothesis-generating rather than definitive proof of mechanism in human disease. We underscore the urgent need for validation through human tissue analysis, cerebrospinal fluid biomarkers, and advanced humanized model systems before Treg-microglia interactions can be established as robust therapeutic targets for AD.

## 1 Introduction

Alzheimer’s disease (AD) accounts for the majority of dementia cases worldwide and arises from a cascade of pathological alterations rather than a single molecular event. Progressive cognitive decline and neuronal degeneration are tightly associated with the accumulation of senile plaques derived from aberrant aggregation of amyloid-beta (Aβ) and neurofibrillary tangles induced by hyperphosphorylation of the microtubule-associated protein tau, ultimately leading to massive neuronal loss and synaptic dysfunction (DeTure and Dickson, 2019; Scheltens et al., 2021).

Notably, these pathological changes begin years to decades before clinical symptoms emerge, transitioning through asymptomatic stages and mild cognitive impairment (MCI) prior to overt dementia. During this preclinical window, specific early stage biomarkers include blood-based phosphorylated tau-181, glial fibrillary acidic protein, and phosphoglycerate dehydrogenase, as well as cerebrospinal fluid (CSF)-based peptide ratios such as Aβ37/Aβ42, which enable detection of pathology prior to irreversible neurodegeneration (Prajapati et al., 2024). Mechanistically, inflammatory cytokines can increase the activity of tau kinases, thereby promoting neurofibrillary tangle formation and establishing a self-perpetuating cycle of neurodegeneration. Multiple signaling pathways drive this neuroinflammatory cascade: the interaction of Aβ oligomers with BBB and glial cell membrane receptors activates inflammatory mediator release, while impaired neuroprotective pathways further compromise the brain’s capacity for tissue repair and regeneration (Parashar et al., 2024). These findings underscore that targeting neuroinflammatory mechanisms, particularly the cellular mediators of innate and adaptive immunity, may offer promising therapeutic avenues for AD intervention.

Central to this neuroinflammatory network are microglia and regulatory T cells (Tregs), which play irreplaceable and interconnected roles in AD pathophysiology. Under physiological conditions, microglia maintain tissue homeostasis by efficiently phagocytosing and clearing aberrantly aggregated Aβ, apoptotic cells, and metabolic waste (Leng and Edison, 2021). However, in the pathological microenvironment of AD, prolonged exposure to Aβ oligomers and aberrant tau proteins drives a phenotypic and functional switch in microglia, shifting these cells from a homeostatic, neuroprotective state toward chronically activated, pro-inflammatory phenotypes that exacerbates neuronal damage. Serving as a critical immunological brake on microglial-driven neuroinflammation are regulatory Tregs, a specialized subset of CD4^+^ T lymphocytes critical for maintaining immune tolerance and suppressing excessive inflammatory responses. Under physiological conditions, Tregs can cross the BBB, inhibit excessive microglial activation through both cell-contact-dependent mechanisms and secretion of anti-inflammatory factors such as transforming growth factor-β (TGF-β) and interleukin-10 (IL-10), thereby driving microglial transition toward pro-resolutive states and enhancing their phagocytic clearance capacity. The disruption of this Treg-microglia regulatory axis in AD creates a permissive environment for sustained neuroinflammation and accelerated disease progression (Abbott et al., 2025).

It is crucial to acknowledge that most mechanistic insights into Treg-microglia interactions derive from transgenic mouse models such as APP/PS1, 5xFAD, and 3xTg-AD. While these models have been invaluable for understanding disease mechanisms, they harbor significant limitations in recapitulating human AD pathology. Murine models typically exhibit early onset, aggressive Aβ deposition driven by familial AD mutations, whereas the vast majority of human AD cases are late-onset and sporadic, developing over decades in the context of aging and metabolic comorbidities. Furthermore, fundamental differences exist between mouse and human immune systems, including microglial transcriptomic profiles, Treg stability under chronic inflammation, and the overarching influence of human aging-associated immune dysfunction (inflammaging). Therefore, throughout this review, we emphasize that findings from rodent studies must be interpreted with caution, and validation through human tissue analysis, cerebrospinal fluid biomarkers, and advanced humanized model systems remains essential for translational relevance.

Despite growing interest in neuroimmunology and AD, previous reviews have largely examined microglia biology, neuroinflammation mechanisms, or adaptive immunity in neurodegenerative diseases as separate entities. This review distinguishes itself by systematically focusing on the bidirectional crosstalk between Tregs and microglia as a unified regulatory axis in AD pathogenesis. We examine the biological characteristics and functional alterations of microglia across different AD stages, then discuss Treg biology and their immunomodulatory roles in the CNS, followed by an in-depth analysis of the molecular mechanisms underlying Treg-microglia interactions. We further critically evaluate the translational challenges posed by current preclinical models and conclude by exploring therapeutic strategies targeting this neuroimmune axis. Many key scientific questions remain unresolved regarding the molecular mechanisms underlying Treg-microglia crosstalk, the critical signaling pathways involved, and their dynamic changes across different stages of AD. In-depth dissection of the interaction between these two cell types will not only refine the theoretical framework of AD immunopathology but also offer novel targets and strategies for the development of immunomodulatory therapies. Collectively, understanding the regulatory network between Tregs and microglia in AD is essential for uncovering the immunopathological basis of the disease and designing effective targeted interventions, representing substantial value in promoting the translational application of basic AD research into clinical practice.

## 2 Microglia as a potential innate-immune hub in Treg-mediated regulation

To contextualize the Treg-microglia crosstalk central to this review, it is necessary to first delineate how microglial phenotypic plasticity determines their responsiveness to Treg-derived signals. This section characterizes microglial states not as isolated entities, but as dynamic interaction partners whose trajectory is increasingly shaped by the presence or absence of adequate Treg regulation.

### 2.1 Basic biological characteristics of microglia

Microglia are the most abundant immune cells in the CNS, accounting for approximately 10–15% of the total central nervous system cells (Nayak et al., 2014). Endowed with unique origins, developmental processes and physiological functions, they serve as the core regulators of the brain’s immune microenvironment. In terms of developmental origin, microglia are not derived from bone marrow hematopoietic stem cells, but arise from primitive macrophages in the embryonic yolk sac (Gomez Perdiguero et al., 2015). These primitive immune cells migrate into the central nervous system and colonize it prior to blood–brain barrier formation, thereby establishing themselves as the resident innate immune population of the CNS. Their development is highly autonomous. Once colonized, microglia maintain and renew their population through self-proliferation and are barely replenished or regulated by peripheral monocytes in adulthood, which fundamentally distinguishes them from peripheral macrophages. At the molecular phenotype level, mature microglia express specific signature markers including TMEM119 and P2RY12, which are relatively specific to microglia compared to other CNS immune populations (Lier et al., 2021). These markers serve as key criteria to distinguish microglia from other immune cell populations in the central nervous system.

Under physiological conditions, microglia exhibit a highly ramified, resting phenotype. Their fine processes continuously extend and survey the surrounding neural microenvironment, performing multiple core physiological functions. First, they conduct immune surveillance: through surface receptors, microglia can detect subtle pathological alterations in the brain, such as cellular damage and abnormal protein aggregation (Hickman et al., 2013). Second, they perform phagocytic and clearance functions, specifically recognizing and engulfing apoptotic neurons, cellular metabolic waste, and abnormally aggregated proteins, thereby maintaining a clean microenvironment in the brain. Third, they mediate synaptic remodeling: during neural development, microglia participate in the formation and pruning of neural circuits by phagocytosing redundant or abnormal synapses, thus regulating the connectivity efficiency of neurons (Paolicelli et al., 2011).

Upon injury or stimulation, microglia rapidly undergo morphological and functional transformation from a homeostatic state to an activated phenotype, initiating immune responses against pathological insults. This activated state is not uniform; microglia can adopt distinct functional phenotypes depending on the nature and severity of the stimulus. For instance, in the context of AD, microglial repopulation has been shown to restore BDNF expression, thereby contributing to synaptic plasticity and cognitive improvement (Wang et al., 2023). This transition between homeostatic and activated states is critically governed by dual regulatory mechanisms. Autocrine TGF-β signaling via the SMAD2/3 pathway maintains the homeostatic microglial signature, characterized by the expression of P2RY12 and TMEM119 (Li et al., 2024). Concurrently, Treg-derived IL-10 suppresses the transition toward pro-inflammatory states (Xie et al., 2015), collectively preserving the neuroprotective phenotype required for efficient clearance of pathological substrates such as Aβ. Together, these properties position microglia as central orchestrators of immune homeostasis, capable of dynamically balancing surveillance, neuroprotection, and inflammatory regulation in response to the changing neural microenvironment. It should be noted that while the fundamental biology of microglia is conserved across species, significant transcriptomic and functional differences exist between murine and human microglia, which may influence the interpretation of findings derived from rodent models.

### 2.2 Microglial heterogeneity and context-specific activation states in AD

The traditional classification of microglial activation into binary M1 and M2 states has proven insufficient to capture the transcriptional and functional complexity observed in AD pathology. Recent single-cell transcriptomic analyses have revealed that microglia exist along a dynamic spectrum of molecular states, with distinct subpopulations emerging in response to specific spatiotemporal cues, genetic background, and disease stage (Paolicelli et al., 2022).

In the context of Aβ pathology, a distinct subpopulation termed disease-associated microglia (DAM) has been identified through single-cell RNA sequencing of AD transgenic mouse models. DAM are characterized by downregulation of homeostatic markers (P2ry12, Cx3cr1, Tmem119) and upregulation of phagocytic and lipid metabolism genes including Apoe, Trem2, Tyrobp, Cst7, and Lpl. Notably, DAM accumulation occurs in two sequential stages: an initial TREM2-independent phase (stage 1) involving downregulation of microglial checkpoint genes, followed by a TREM2-dependent phase (stage 2) marked by enhanced expression of phagocytic machinery. These cells localize specifically to Aβ plaques and display amoeboid morphology with intracellular phagocytic Aβ particles, suggesting a dedicated role in plaque clearance and restriction of neurodegeneration (Keren-Shaul et al., 2017). Concurrently, activation of the TREM2-APOE signaling pathway drives a transition from homeostatic microglia to a microglial neurodegenerative phenotype (MGnD). This transcriptional program is conserved across multiple neurodegenerative diseases including AD, amyotrophic lateral sclerosis, and multiple sclerosis. The APOE-mediated pathway suppresses homeostatic transcription factors while inducing genes involved in phagocytosis of apoptotic neurons and lipid metabolism. Importantly, MGnD microglia exhibit impaired tolerogenic functions and lose the ability to suppress the proliferation of adaptive immune cells, such as T cells, thereby disrupting immune homeostasis in the brain (Krasemann et al., 2017).

Beyond DAM and MGnD, integrative analyses of aging and AD risk factors have revealed additional microglial states with distinct functional implications. Other studies have identified two major activated microglia populations in response to Aβ accumulation: activated response microglia (ARMs) and interferon-response microglia (IRMs). ARMs express major histocompatibility complex class II molecules and putative tissue repair genes (Dkk2, Gpnmb, Spp1), and show strong enrichment for AD genetic risk loci. In contrast, IRMs display an interferon-signature profile independent of APOE genotype. Notably, the transition toward ARMs is accelerated by aging and female sex, whereas APOE4 genotype specifically modulates the ARMs trajectory without affecting IRMs, demonstrating that genetic and environmental risk factors converge on distinct microglial activation programs (Sala Frigerio et al., 2019). The functional implications of this heterogeneity are context-dependent and temporally dynamic. Microglial states exhibit remarkable plasticity, transitioning between homeostatic surveillance, phagocytic clearance, and inflammatory responses based on local microenvironmental cues. Instead, microglia adopt specialized states that may simultaneously express both inflammatory mediators and phagocytic receptors, with their net effect on neurodegeneration determined by the balance of these functions and their spatial relationship to pathology. For the purpose of clarity in subsequent sections, we will refer to microglial states characterized by high expression of pro-inflammatory cytokines (e.g., IL-1β, TNF-α) and compromised phagocytosis as pro-inflammatory reactive microglia, and states associated with enhanced Aβ clearance and pro-resolutive cytokine production (e.g., IL-10, TGF-β responsive) as pro-resolutive reactive microglia, acknowledging that these represent simplifications of the complex spectra described above (DAM, MGnD, ARMs). However, it is important to recognize that the disease-associated microglial signatures identified in transgenic mouse models only partially overlap with those observed in human AD brains, underscoring the need for cautious extrapolation of these phenotypic classifications to human pathophysiology.

### 2.3 The relationship between microglial dysfunction and AD

Microglial dysfunction is a central trigger for neuroinflammation and neuronal degeneration in Alzheimer’s disease. Its functional abnormalities mainly manifest in two aspects: disordered inflammatory regulation and impaired phagocytic clearance, which together form a vicious “inflammation–damage” cycle during AD pathogenesis (Heneka et al., 2015). In terms of inflammatory regulation, microglia under AD pathological conditions undergo persistent pro-inflammatory activation, secreting large amounts of pro-inflammatory cytokines including tumor necrosis factor-α (TNF-α), interleukin-1β (IL-1β), and interleukin-6 (IL-6). They also activate the NLRP3 inflammasome, further amplifying neuroinflammatory responses in the brain (Halle et al., 2008; Heneka et al., 2013; Kinney et al., 2018). These pro-inflammatory factors directly damage neuronal membranes and synaptic structures, leading to reduced synaptic plasticity and abnormal neuronal excitability, ultimately resulting in synaptic loss and neuronal apoptosis (Ising and Heneka, 2018). Meanwhile, the pro-inflammatory microenvironment also disrupts the integrity of the BBB, allowing peripheral immune cells to infiltrate the brain, further exacerbating central neuroinflammation and amplifying neuronal damage (Zenaro et al., 2015).

With regard to phagocytic clearance, microglial dysfunction is characterized by a marked reduction in the phagocytosis of substrates such as Aβ and apoptotic neurons, a phenomenon closely associated with abnormalities in the TREM2-APOE signaling pathway (Yeh et al., 2016; Krasemann et al., 2017; Ulland et al., 2017). Under physiological conditions, microglia recognize Aβ via various surface receptors including TREM2 and CD36, initiate phagocytic signaling pathways, internalize Aβ, and degrade it through the lysosomal pathway (El Khoury et al., 2003; Mueller-Steiner et al., 2006; Zhao et al., 2018; Li et al., 2022). The TREM2-APOE signaling pathway is a core pathway regulating the Aβ phagocytic function of microglia. Upon binding to Aβ, TREM2 activates the downstream PI3K-Akt signaling, promoting the recruitment of microglia to Aβ plaques and enhancing their phagocytic capacity (Krasemann et al., 2017; Ulland and Colonna, 2018). APOE acts as a ligand that binds to TREM2, further amplifying this signal and improving the efficiency of Aβ clearance (Yeh et al., 2016). Microglia can also secrete proteases such as insulin-degrading enzyme and neutral endopeptidase, which directly degrade Aβ oligomers extracellularly and prevent their aggregation into plaques (Hickman et al., 2008; Tamboli et al., 2010). Impaired microglial phagocytosis not only leads to the continuous accumulation of Aβ plaques, but also prevents the timely clearance of apoptotic neurons and metabolic waste in the brain, further aggravating neurodegeneration and ultimately resulting in progressive cognitive decline. Studies have confirmed that the degree of microglial dysfunction is significantly positively correlated with the severity of cognitive impairment in AD mice. Therapeutic strategies that enhance TREM2 function and activate CSF1R are effective in restoring normal microglial function, and can alleviate neuroinflammation and cognitive damage in AD mice (Olmos-Alonso et al., 2016; Schlepckow et al., 2020). Oxidative stress and disturbed lipid metabolism in the AD brain alter the metabolic phenotype of microglia, shifting them from oxidative phosphorylation to glycolysis. This metabolic reprogramming further suppresses microglial phagocytosis, reduces Aβ clearance efficiency, and triggers a vicious cycle in AD (Marschallinger et al., 2020; Nugent et al., 2020) . These mechanistic insights, while valuable, are predominantly derived from rodent studies, and the temporal dynamics of microglial dysfunction in the context of human aging and decades-long disease progression may differ substantially.

## 3 Tregs as adaptive immune modulators: shaping microglial function in AD

While microglia serve as the primary effectors of neuroinflammation, their functional trajectory in AD is profoundly shaped by Treg-mediated immune regulation. This section examines Treg biology specifically through the lens of how these cells lose the capacity to maintain microglial homeostasis as AD progresses.

### 3.1 Basic biological characteristics of Tregs

Tregs are a subset of CD4^+^ T cells characterized by the core phenotype CD4^+^CD25^+^FOXP3^+^. Based on their origin, they can be divided into naturally occurring thymus-derived Tregs (nTregs) and peripherally induced adaptive Tregs (pTregs), which jointly maintain immune tolerance and homeostasis. FOXP3 serves as the lineage-specific transcription factor for Tregs and directly governs their differentiation, proliferation, and functional stabilities. Loss of FOXP3 expression results in complete functional ablation of Tregs and leads to severe systemic autoimmune diseases (Georgiev et al., 2024).

The immunoregulatory mechanisms of Tregs mainly fall into two categories: cell-contact-dependent and cytokine-dependent pathways, which act in concert to exert immunosuppressive effects. In the cell-contact-dependent mechanism, molecules expressed on Tregs—such as cytotoxic T-lymphocyte-associated antigen 4 (CTLA-4), programmed cell death protein 1 (PD-1), and lymphocyte activation gene 3 (LAG-3)—mediate suppression via distinct ligand interactions: CTLA-4 binds to CD80/CD86 on antigen-presenting cells (APCs), leading to their downregulation or internalization; PD-1 engages PD-L1/PD-L2 to inhibit effector T cell function; and LAG-3 interacts with MHC-II molecules to dampen dendritic cell activation (Yan et al., 2022). Through competitive binding or inhibitory signaling, these interactions directly suppress the activation and proliferation of effector T cells, macrophages, and other immune cells. In the cytokine-dependent pathway, Tregs secrete pro-resolutive cytokines including IL-10, TGF-β, and IL-35, creating an immunosuppressive local microenvironment that nonspecifically restrains excessive immune responses (Gozálvez et al., 2025).

Tregs play an irreplaceable role in maintaining immune tolerance. They can suppress the activation and proliferation of autoreactive T cells, thereby preventing the development of autoimmune diseases. Tregs also modulate the magnitude of immune responses following pathogen infection, preventing excessive inflammation from damaging healthy tissues. Tregs also participate in tissue repair following injury, sustaining tissue homeostasis by suppressing inflammation and facilitating tissue regeneration (Dombrowski et al., 2017). Within the central nervous system, a small number of Tregs can traverse the blood–brain barrier (BBB) under physiological conditions, contributing to central immune tolerance and restraining pathological neuroinflammation (Xie et al., 2015).

### 3.2 Changes in Treg number, phenotype, and function in AD: an unresolved and contradictory landscape

A growing body of literature has investigated alterations in Treg frequency, phenotype, and suppressive function in the context of AD. However, it must be emphasized at the outset that the existing human data are far from consistent, with considerable discrepancies across studies likely attributable to differences in disease stage, sample source, patient cohort characteristics, and methodological approaches for Treg identification and functional assessment.

#### 3.2.1 Contradictory findings on Treg frequency in AD patients

Studies have reported that an increase in the proportion of circulating CD4^+^CD25^+^FoxP3^+^ Tregs in the peripheral blood of AD patients compared with age-matched healthy controls (Saresella et al., 2010). In contrast, another investigation has found a decrease or no significant change in peripheral Treg frequencies in AD cohorts (Larbi et al., 2009). These conflicting results underscore the complexity of peripheral immune remodeling in AD and caution against drawing uniform conclusions about the directionality of Treg frequency changes. The heterogeneity of findings may partly reflect methodological variation: Saresella et al. defined Tregs by intracellular FoxP3 staining and examined patients across the full disease spectrum (MCI to severe AD), whereas Larbi et al. relied solely on surface CD25 expression and restricted their cohort to mild AD only.

#### 3.2.2 Conflicting data on Treg suppressive function

Functional assessments of Tregs in AD have yielded discordant results. One report demonstrated that Tregs isolated from moderate-to-severe AD patients exhibit impaired suppressive capacity in co-culture assays (Faridar et al., 2020), associated with reduced CD25 expression. Conversely, Rosenkranz et al. reported enhanced suppressive activity in AD patients (Rosenkranz et al., 2007), while Saresella et al. observed augmented suppression specifically in MCI but not in established AD dementia (Saresella et al., 2010). Notably, Oberstein et al. found unaltered Treg proportions across disease stages but did not assess functional suppression (Oberstein et al., 2018). These discrepancies suggest Treg function may vary by disease stage and methodological approach, requiring dynamic characterization.

#### 3.2.3 Treg trafficking into the CNS: limited and largely indirect evidence

Whether Tregs physically infiltrate the AD brain parenchyma in significant numbers remains an open and largely unresolved question. While Baruch et al. demonstrated that transient Treg depletion leads to subsequent accumulation of FoxP3^+^ cells in murine AD brain parenchyma (Baruch et al., 2015); studies in human AD brain tissue have primarily identified CD3^+^CD8+ cytotoxic T cells, while FoxP3^+^ Tregs remain largely uncharacterized or unassessed in human parenchyma (Merlini et al., 2018). Most of the evidence for Treg-mediated neuroprotection or immunomodulation in the CNS derives from peripheral observations or from murine models, where the translatability to human AD remains uncertain. It is therefore important to distinguish between Treg-mediated effects that may occur through peripheral immune modulation and those that require direct Treg presence within the CNS parenchyma—the latter scenario being far less well-supported by current evidence.

#### 3.2.4 The biphasic hypothesis: a plausible but unverified framework

Some investigators have proposed a biphasic model of Treg involvement in AD. According to this hypothesis, Tregs may exert beneficial, pro-resolutive effects at early disease stages—when dampening excessive microglial activation and limiting collateral neuronal damage is advantageous—but become detrimental at later stages, when their immunosuppressive activity may impede the clearance of Aβ plaques by phagocytic microglia and infiltrating immune cells (Dansokho et al., 2016). This framework provides an intellectually attractive explanation for the otherwise conflicting experimental results: transient Treg depletion in late-stage AD mouse models (e.g., 5xFAD) has been associated with subsequent Aβ clearance and cognitive improvement (Baruch et al., 2015), whereas Treg expansion or adoptive transfer in early stage models has shown neuroprotective effects (Baek et al., 2016; Dansokho et al., 2016).

However, it is essential to note that this biphasic model remains an unverified hypothesis rather than an accepted conclusion. The supporting evidence is derived almost exclusively from murine models that imperfectly recapitulate human AD pathology. Most AD transgenic mice develop aggressive amyloidosis without the full spectrum of tau pathology, neuronal loss patterns, and vascular changes observed in human disease (Drummond and Wisniewski, 2017). Furthermore, the timing of Treg manipulation in these models (depletion versus expansion at defined ages) is difficult to map onto clinical disease stages in patients. The biphasic concept therefore represents a working hypothesis that requires rigorous validation through longitudinal studies in human cohorts and through the development of more faithful animal models.

To provide a consolidated overview of the current state of evidence, Table 1 summarizes the key findings from representative human studies regarding Treg frequency, phenotype, and function in AD, explicitly highlighting the methodological differences and conflicting data that contribute to this unresolved landscape.

**TABLE 1:** Summary of human studies on regulatory T-cell alterations in Alzheimer’s disease: discrepancies in frequency, phenotype, and suppressive function.

Controversial aspect	Sample source	Disease stage	Methodological approach	Key findings	Study
Treg frequency	Peripheral blood	MCI to severe AD	Flow cytometry; intracellular FoxP3 staining; CD4^+^CD25^+^FoxP3^+^	Increased total Treg proportion in MCI and AD; potent PD1neg subset increased only in MCI compared to age-matched healthy controls	(Saresella et al., 2010)
	Peripheral blood	Mild AD only	Flow cytometry; surface CD25 staining only; CD4^+^CD25^+^	Significantly decreased Treg frequency	(Larbi et al., 2009)
	Peripheral blood (cryopreserved PBMCs)	MCI, mild, moderate, severe AD	Flow cytometry; CD4^+^CD127lowCD25^+^ (surface markers); no intracellular FoxP3 staining	Unaltered Treg proportions across all disease stages (AD dementia, MCI_AD, MCI_other vs. controls); Treg frequency positively correlated with total Tau and pTau181 in AD group	(Oberstein et al., 2018)
	Peripheral blood	Mild to moderate AD	Flow cytometry; CD4^+^/CD25high/CD127low-neg (surface markers, non-fixed cells); subtyped as Resting/Activated/Secreting	Significantly reduced total Treg frequency in AD vs. controls (*P* = 0.0320); significantly reduced resting Treg subset (*P* < 0.0001); correlation with cognitive scores not assessed	(Ciccocioppo et al., 2019)
Treg phenotype	Peripheral blood	Moderate-to-severe AD	Flow cytometry; CD25 surface expression intensity on CD4^+^ Tregs	Reduced CD25 expression on Tregs, suggesting phenotypic instability in late-stage AD	(Faridar et al., 2020)
Treg function	Peripheral blood	AD (stage not clearly delineated)	CD4^+^CD25^+^ Tregs; co-culture suppression assay	Exhibited enhanced suppressive activity relative to young controls, with a trend toward increased activity compared to age-matched elderly controls	(Rosenkranz et al., 2007)
	Peripheral blood	MCI to severe AD	CD4^+^CD25^+^FoxP3^+^; co-culture suppression assay	Augmented suppressive function in MCI; suppression not enhanced in established AD dementia	(Saresella et al., 2010)
	Peripheral blood	Moderate-to-severe AD	CD4^+^CD25^+^ Tregs; co-culture suppression assay	Impaired suppressive capacity; associated with reduced CD25 expression	(Faridar et al., 2020)

## 4 Molecular mechanisms of Treg–microglia crosstalk in AD

### 4.1 Cytokine-mediated regulation

#### 4.1.1 The IL-10 pathway

IL-10 is a pleiotropic pro-resolutive cytokine and one of the principal effector molecules through which Tregs exert immunosuppressive functions. In the CNS, IL-10 acts on microglia primarily through binding to the IL-10 receptor (IL-10R), a heterodimer composed of IL-10Rα and IL-10Rβ subunits, which is constitutively expressed on the microglial surface (Lobo-Silva et al., 2016). Upon ligand engagement, IL-10R activates the JAK1/TYK2 signaling cascade, leading to the phosphorylation and nuclear translocation of STAT3. This signaling axis is essential for IL-10’s pro-resolutive functions, including the suppression of pro-inflammatory mediators such as TNF-α, IL-1β, and nitric oxide (Moore et al., 2001; Saraiva and O’Garra, 2010).

Evidence supporting the functional relevance of Treg-derived IL-10 in modulating microglial activity has been provided by studies in normal CNS tissue. Xie et al. demonstrated that cerebral Tregs restrain microglia/macrophage-mediated inflammatory responses via IL-10, using both *in vitro* co-culture systems and *in vivo* studies in the normal rat cerebrum (Xie et al., 2015). In their co-culture experiments, Tregs suppressed lipopolysaccharide (LPS)-induced microglial activation in an IL-10-dependent manner, as neutralization of IL-10 significantly abrogated the suppressive effect of Tregs on microglial TNF-α and IL-6 production. These findings establish a mechanistic link between Treg-secreted IL-10 and the attenuation of microglial pro-inflammatory activation, though validation in AD-specific models remains necessary. Thus, maintaining physiological Treg function is critical for preventing neurotoxic microglial hyperactivation.

Notably, the role of IL-10 in AD is not unequivocally beneficial and presents a complex double-edged sword. Studies have shown that adeno-associated virus (AAV)-mediated overexpression of IL-10 in the brains of APP transgenic mice paradoxically increased Aβ plaque burden and worsened cognitive deficits, likely by excessive suppression of the microglial activation state required for phagocytic clearance (Chakrabarty et al., 2015). Corroborating this, it has been demonstrated that IL-10 genetic deficiency in APP/PS1 mice reduces IL-10/STAT3 signaling in microglia, thereby releasing the brake on microglial Aβ phagocytosis and mitigating cerebral amyloidosis (Guillot-Sestier et al., 2015).

However, these findings must be reconciled with the detrimental consequences of severe immune dysregulation seen in later disease stages. As AD progresses to intermediate and advanced stages, the declining number and functional impairment of Tregs result in diminished IL-10 availability in the CNS microenvironment. This loss of Treg-mediated homeostatic regulation contributes to an unabated shift toward a sustained pro-inflammatory, neurotoxic microglial phenotype (Faridar et al., 2020). Therefore, a delicate temporal and spatial balance emerges: while supraphysiological IL-10 levels (or basal levels in an amyloid-rich environment) may paradoxically suppress the microglial phagocytic capacity and exacerbate amyloid pathology, the chronic loss of Treg-derived IL-10 accelerates inflammatory neuronal damage. This dual nature underscores the importance of maintaining IL-10 within an optimal therapeutic window rather than pursuing its blanket augmentation or complete ablation.

#### 4.1.2 The TGF-β pathway

TGF-β is another key immunosuppressive cytokine constitutively produced by Tregs and is critically involved in both Treg differentiation and effector function. In the CNS, TGF-β signaling plays a fundamental role in maintaining microglial homeostasis. TGF-β serves as an essential and non-redundant factor for the development and maintenance of the unique homeostatic molecular signature of microglia, including the expression of P2ry12, Tmem119, and Sall1. Disruption of TGF-β signaling in microglia leads to the loss of this homeostatic identity and the acquisition of a neurodegenerative phenotype (Li et al., 2024).

Mechanistically, TGF-β signals through a heteromeric complex of type I (TGF-βRI/ALK5) and TGF-βRII serine/threonine kinase receptors expressed on microglia. Ligand binding triggers phosphorylation of the canonical SMAD2/3 transcription factors, which then form a complex with SMAD4 and translocate to the nucleus to regulate target gene expression (Chia et al., 2024). In microglia, activation of the SMAD2/3 pathway suppresses NF-κB-dependent pro-inflammatory gene transcription (such as iNOS and IL-6) and promotes the expression of alternative activation markers associated with neuroprotection (Spittau et al., 2013). Additionally, TGF-β can signal through non-canonical pathways, including TAK1-mediated activation of p38 MAPK and JNK, which may modulate microglial migration and phagocytosis (Zhang, 2017).

In the context of AD, the role of TGF-β signaling is complex and multifaceted. Reduced TGF-β type II receptor expression in neurons of AD transgenic mice accelerated neurodegeneration and promoted Aβ accumulation, suggesting a neuroprotective role for intact TGF-β signaling (Tesseur et al., 2006). Blocking TGF-β/SMAD signaling specifically in peripheral macrophages enhanced their infiltration into the brain and promoted Aβ clearance, indicating that the effects of TGF-β are cell-type-specific and context-dependent (Town et al., 2008). Regarding the microglia axis specifically, TGF-β signaling is essential for inducing Aβ phagocytosis while suppressing the production of neurotoxic reactive oxygen species and nitric oxide. However, this protective TGF-β1-Smad3 functional response becomes severely impaired with aging, shifting microglia toward a cytotoxic phenotype (Tichauer et al., 2014).

During AD progression, TGF-β signaling undergoes significant alterations on both the receptor and ligand fronts. In early stages, Treg-derived TGF-β contributes to maintaining microglial homeostasis and supporting their surveillance and phagocytic functions. As the disease advances, however, chronic neuroinflammation and the accumulation of pathological proteins impair TGF-β signaling in microglia. DAM in AD exhibit a TREM2-APOE pathway-driven suppression of homeostatic genes, including downregulated TGF-β receptor 1 (Tgfbr1) expression, rendering them less responsive to TGF-β-mediated regulation (Krasemann et al., 2017). Concurrently, the functional decline of Tregs in advanced AD further reduces the availability of TGF-β in the CNS microenvironment (Faridar et al., 2020). As demonstrated in Treg-depletion models, this loss of Treg-mediated immunosuppression creates a feedforward loop that perpetuates microglial dysregulation, exacerbates amyloid pathology, and accelerates cognitive deficits (Baek et al., 2016). While direct evidence specifically linking Treg-derived TGF-β to microglial modulation in AD remains limited, extrapolation from peripheral and other CNS disease models provides a reasonable mechanistic framework.

#### 4.1.3 The IL-35 pathway

IL-35, a heterodimeric cytokine composed of p35 and Ebi3 subunits, is preferentially secreted by Tregs (Collison et al., 2007). Direct studies on microglia in AD remain scarce, but Luo et al. demonstrated in a type 1 diabetes model that IL-35 directly suppressed pro-inflammatory cytokine production in sorted CD11b^+^F4/80^+^ macrophages independent of lymphocyte-mediated secondary effects (Luo et al., 2022), suggesting a potential for similar myeloid cell modulation in the CNS.

Importantly, contemporary understanding of microglial activation has moved beyond the simplistic M1/M2 dichotomy. Single-cell transcriptomic studies reveal that microglial states in AD encompass a spectrum of context-dependent phenotypes, including disease-associated microglia and various intermediate reactive states (He et al., 2025). How Treg-derived cytokines differentially affect these distinct microglial subpopulations, and whether they reach sufficient local concentrations in the CNS parenchyma to exert meaningful effects, remain open questions requiring direct investigation.

### 4.2 Contact-dependent regulation via co-signaling molecules

#### 4.2.1 The CTLA-4/CD80-CD86 axis

CTLA-4 is constitutively expressed on Tregs and serves as a critical mediator of their suppressive function. CTLA-4 binds with high affinity to the co-stimulatory ligands CD80 (B7-1) and CD86 (B7-2), which are expressed on antigen-presenting cells, including activated microglia in the CNS (Louveau et al., 2015). The mechanisms through which CTLA-4 modulates target cell function include competitive inhibition of CD28-mediated co-stimulation, trans-endocytosis of CD80/CD86 from the surface of antigen-presenting cells, and induction of indoleamine 2,3-dioxygenase (IDO) expression (Mellor et al., 2004; Qureshi et al., 2011; Walker and Sansom, 2015).

In the peripheral immune system, the CTLA-4/CD80-CD86 axis is one of the best-characterized mechanisms of Treg-mediated suppression. Qureshi et al. (2011) demonstrated that CTLA-4 physically captures and removes CD80 and CD86 from opposing cells through trans-endocytosis, thereby depleting co-stimulatory ligands and rendering target cells less capable of activating effector T cells (Qureshi et al., 2011). This mechanism has been extensively validated in dendritic cells and macrophages.

However, direct evidence for CTLA-4-mediated regulation of microglia by Tregs within the AD brain parenchyma remains limited. The plausibility of this interaction is supported by several lines of indirect evidence. First, although *in vitro* and animal models demonstrate clear CD86 upregulation on activated microglia (Ponomarev et al., 2005), detecting robust CD80 and CD86 expression on human microglia in postmortem AD brains has proven challenging, highlighting the complex and dynamic nature of microglial phenotypes *in vivo* (Walker and Lue, 2015). Whether microglia in the AD brain express sufficient CD80/CD86 to engage this CTLA-4-mediated regulatory mechanism remains an area requiring further investigation. Second, Tregs that infiltrate the CNS following neurological injuries display specialized tissue-resident phenotypes and retain strong suppressive capacity (Ito et al., 2019). Third, adoptive transfer of ex vivo expanded human Tregs—which highly express contact-dependent co-inhibitory molecules—has been shown to significantly downregulate microglial activation markers (such as Trem2 and Tyrobp) and reduce pro-inflammatory gene networks in the brains of 5xFAD mice (Faridar et al., 2022).

Nevertheless, formal demonstration that Treg-expressed CTLA-4 directly engages CD80/CD86 on microglia in situ and modulates their functional phenotype via trans-endocytosis or other contact-dependent mechanisms in AD models is still lacking. Future studies employing microglia-specific CD80/CD86 conditional knockout mice crossed with AD transgenic models, or advanced two-photon imaging techniques to visualize Treg–microglia immune synapses *in vivo*, are needed to establish this pathway definitively.

#### 4.2.2 The PD-1/PD-L1 axis

PD-1 is an inhibitory receptor expressed on activated T cells, including Tregs, while its ligand PD-L1 (B7-H1) is expressed on various cell types. The PD-1/PD-L1 axis has garnered considerable attention in AD research following the provocative findings of Baruch et al. who reported that PD-1 immune checkpoint blockade reduced cerebral amyloid plaque load and improved cognitive performance in 5xFAD transgenic mice (Baruch et al., 2016). The authors proposed that PD-1 blockade enhanced the recruitment of monocyte-derived macrophages to the CNS, which facilitated Aβ clearance.

However, subsequent studies have yielded conflicting results, casting doubt on the generalizability of these findings. Latta-Mahieu et al. failed to replicate the beneficial effects of anti-PD-1 treatment in multiple AD mouse models (Latta-Mahieu et al., 2018), and Obst et al. (2018) similarly reported that genetic deletion of PD-1 in a prion disease model of chronic neurodegeneration did not induce myeloid mobilization to the brain or significantly alter the neuroinflammatory profile, though a slight exacerbation of behavioral deficits was observed (Obst et al., 2018). These discrepancies may reflect differences in experimental protocols, mouse genetic backgrounds, or the timing of intervention relative to disease stage. Beyond the question of whether systemic PD-1 blockade is therapeutically beneficial, an important mechanistic question concerns how specific cell populations in the AD brain interact through the PD-1/PD-L1 axis.

Regarding the specific role of PD-1/PD-L1 in Treg–microglia interactions, the evidence remains largely inferential. Recent findings show a complex expression pattern in the AD brain: while microglia upregulate PD-1 in response to amyloid pathology (Kummer et al., 2021), they can also express PD-L1 under inflammatory conditions. Therefore, the PD-1/PD-L1 engagement between Tregs and microglia could theoretically modulate both cell types via complex bidirectional signaling. On the Treg side, PD-1 signaling may promote Treg stability and suppressive function (Francisco et al., 2010); on the microglial side, either direct engagement of microglial PD-1 or constitutive intrinsic signaling by PD-L1 could attenuate their pro-inflammatory activation and phagocytic dysfunction (Hartley et al., 2018; Kummer et al., 2021). Although these findings derive primarily from peripheral macrophages and tumor contexts, microglia share substantial functional overlap with macrophages, and similar PD-1/PD-L1 regulatory mechanisms likely operate at the Treg–microglia interface in the AD brain. However, whether this precise bidirectional signaling occurs between Tregs and microglia in the AD brain and what functional consequences it carries remain open questions that require direct experimental validation.

### 4.3 Chemokine-mediated Treg recruitment and positioning

The recruitment of Tregs to the CNS is a prerequisite for their local immunoregulatory function, and this process is governed by chemokine–chemokine receptor axes that guide Treg migration across CNS barriers and within the parenchyma.

CCL2/CCR2 axis: indirect regulation of Treg accumulation. The CCL2/CCR2 axis is a major pathway for monocyte recruitment to the inflamed CNS. In a tauopathy model, Ben-Yehuda et al. (2021) demonstrated that blocking CCR2 abrogated the therapeutic benefit of anti-PD-L1 immunotherapy and completely prevented the accumulation of Tregs in the brain (Ben-Yehuda et al., 2021). Critically, however, the accumulated Tregs themselves expressed only negligible levels of CCR2, and systemic CCR2 blockade did not reduce circulating Treg numbers. These findings indicate that Treg accumulation in the diseased brain is not mediated by direct CCR2-dependent migration, but is instead indirectly controlled through CCR2-dependent monocyte-derived macrophages, which may create a permissive parenchymal milieu for subsequent Treg homing, local proliferation, or in situ conversion from infiltrating CD4^+^ T cells.

CCR6/CCL20 axis: a gateway through the choroid plexus. CCR6 and its ligand CCL20 constitute a critical axis for immune cell entry into the uninflamed CNS. Studies have shown that CCL20 is constitutively expressed by choroid plexus epithelial cells in both mice and humans, and that CCR6-dependent migration through the choroid plexus is essential for the initial entry of Th17 cells into the CNS during EAE (Reboldi et al., 2009). Importantly, CCR6 is also required for Treg recruitment to the CNS. In CCR6-deficient mice, Foxp3^+^ cell infiltration into the spinal cord was reduced by approximately 75% at the peak phase of EAE, resulting in an elevated effector-to-Treg ratio and a more severe, prolonged disease course. Competitive adoptive transfer experiments confirmed that CCR6-deficient Tregs were significantly impaired in their ability to reach CNS target tissues compared to wild-type counterparts. Transfer of wild-type Tregs to CCR6-deficient mice restored the cytokine balance in the CNS and ameliorated disease severity (Villares et al., 2009). These data establish CCR6 as a functional requirement for Treg access to the inflamed CNS. While these findings derive from EAE, a model of acute autoimmune neuroinflammation, the CCR6/CCL20 axis may similarly regulate Treg entry in the chronic inflammatory context of AD, although direct validation is required.

CX3CL1/CX3CR1 axis: neuron–microglia communication and engineered Treg homing. The CX3CL1/CX3CR1 axis primarily mediates neuron–microglia communication in the CNS. CX3CL1, expressed by neurons, signals through CX3CR1 on microglia to maintain their homeostatic state; loss of this signaling in AD models results in impaired microglial plaque engagement, skewing toward a neurodegenerative phenotype, and aggravated cognitive decline (Puntambekar et al., 2022). While endogenous Treg expression of CX3CR1 in the context of AD has not been well characterized, retroviral transduction of CX3CR1 into Tregs significantly enhanced their homing to the brain, particularly the hippocampus, in both LPS-induced neuroinflammation and 3xTg-AD models (Yang et al., 2024). These CX3CR1-transduced Tregs suppressed disease-associated microglial activation, reduced Aβ accumulation, and improved cognitive function more effectively than control Tregs. This engineered approach provides proof-of-concept that the CX3CL1/CX3CR1 axis can be exploited to direct Tregs to sites of neuroinflammation, though whether this represents a physiologically relevant endogenous recruitment mechanism for Tregs in AD remains to be determined. Clinical translation of this genetic engineering approach would require addressing challenges including cell manufacturing scalability, long-term safety of transgene expression, and potential immunogenicity.

CXCL12/CXCR4 axis: tissue-specific Treg positioning. CXCL12/CXCR4 signaling has emerged as a regulator of Treg positioning within the CNS. Through scRNA-seq analysis, Watanabe et al. (2024) found that brain-derived Tregs in EAE preferentially express Cxcr4, in contrast to spinal cord Tregs which express Ccr1, Ccr2, and Cxcr6 (Watanabe et al., 2024). This differential chemokine receptor expression correlated with distinct chemokine environments in the two tissues and influenced tissue-specific Treg localization and function. In the broader context of CNS inflammation, CXCL12 is expressed by BBB endothelial cells and choroid plexus cells, and its redistribution from the basolateral to the luminal surface of brain endothelial vessels has been associated with disease onset in MS, potentially altering immune cell entry into the parenchyma (Cruz-Orengo et al., 2011). Whether similar CXCL12 redistribution occurs in AD and affects Treg trafficking remains an open question.

Choroid plexus as an immunological interface. The choroid plexus serves as a key interface for CNS immune surveillance. The choroid plexus epithelium is constitutively populated by CNS-specific effector memory CD4^+^ T cells (Baruch et al., 2013). With aging, the local cytokine balance at the choroid plexus shifts toward a Th2-dominated profile with elevated IL-4 and reduced IFN-γ, triggering the production of CCL11, a chemokine associated with cognitive dysfunction. This age-related immunosenescence at the choroid plexus may alter the local chemokine milieu and impair the recruitment or functional activation of Tregs, though direct evidence linking choroid plexus dysfunction to diminished Treg entry in AD specifically requires further investigation.

It should be noted that the precise chemokine signals that specifically recruit Tregs, as opposed to other T cell subsets, to the AD brain remain incompletely defined. Many chemokine receptors are shared across different T cell populations, and much of the current evidence derives from EAE or acute neuroinflammation models rather than from AD itself. Single-cell transcriptomic profiling of CNS-infiltrating Tregs in AD models, combined with chemokine receptor-specific conditional knockout approaches, will be essential to delineate Treg-specific recruitment mechanisms in the context of chronic neurodegeneration.

### 4.4 Metabolic microenvironment regulation

#### 4.4.1 Tryptophan-kynurenine metabolism

Tryptophan depletion by IDO creates a metabolically restrictive environment that suppresses effector T cell proliferation. Notably, downstream kynurenine metabolites can activate the aryl hydrocarbon receptor (AhR), which has been shown to promote Treg differentiation and function, suggesting a potential feedback mechanism whereby IDO-driven tryptophan catabolism reinforces local immunosuppression (Mezrich et al., 2010). Concurrently, kynurenine pathway metabolites exert direct effects on microglia. Kynurenic acid, a neuroprotective metabolite, antagonizes NMDA and α7 nicotinic acetylcholine receptors and has been shown to possess pro-resolutive and antioxidant properties (Lugo-Huitrón et al., 2011). Conversely, quinolinic acid, a neurotoxic downstream metabolite produced predominantly by activated microglia, acts as an NMDA receptor agonist and promotes excitotoxicity and oxidative stress (Guillemin et al., 2005).

In AD, the balance between neuroprotective and neurotoxic branches of the kynurenine pathway appears to be dysregulated, though the nature of this dysregulation is more complex than previously assumed. A recent systematic review and meta-analysis reported altered levels of multiple KP metabolites in AD patients, with findings that challenge simple linear models of neurotoxic accumulation and instead suggest stage-specific or compartment-specific metabolic shifts (Choe et al., 2025). In this context, neuronal damage may arise not from absolute excess of quinolinic acid—which systematic reviews and meta-analyses have surprisingly shown to be decreased rather than elevated in AD cerebrospinal fluid—but rather from insufficient compensatory upregulation of neuroprotective metabolites like kynurenic acid, whose elevated CSF levels have been associated with slower disease progression, suggesting complex adaptive mechanisms within the kynurenine pathway (Knapskog et al., 2023).

The concurrent decline in Treg function observed in AD may further modulate this metabolic landscape by disrupting the immune-regulatory mechanisms that normally control IDO activity. Although IDO is upregulated in AD brains—likely driven by chronic neuroinflammation—the immunosuppressive capacity of this upregulation, which normally depends on Treg-mediated CTLA-4 signaling to sustain a tolerogenic kynurenine milieu (as demonstrated in peripheral immune contexts by Fallarino et al. (2003) and Mellor et al. (2004), may be compromised when Treg function is impaired. Recent evidence demonstrates that Treg dysfunction occurs in AD and that adoptive transfer of expanded Tregs can ameliorate neuroinflammation in preclinical models (Faridar et al., 2022). This distinction between inflammatory IDO upregulation (driving neurotoxic metabolite production) and Treg-mediated IDO induction (promoting immunosuppressive tryptophan catabolism) is critical to understanding the dual role of the kynurenine pathway in AD pathology. Nevertheless, direct evidence linking Treg-induced IDO activity to microglial kynurenine metabolism specifically in AD models remains currently lacking, and this framework requires further experimental validation.

#### 4.4.2 mTOR signaling and mitochondrial metabolism

The mTOR signaling pathway serves as a central metabolic hub that differentially regulates Treg and microglial function in neurodegenerative contexts. In Tregs, tight control of mTOR activity is essential: while nutrient-fueled mTORC1 activation supports proliferation and effector function, inappropriate hyperactivation disrupts metabolic quiescence and destabilizes Foxp3 expression, impairing suppressive capacity (Shi and Chi, 2019). In microglia, mitochondrial dysfunction—characterized by the release of oxidized mitochondrial DNA (ox-mtDNA)—activates the NLRP3 inflammasome, driving pro-inflammatory IL-1β release; notably, this mechanism was characterized in Parkinson’s disease models (Gan et al., 2026) and requires validation in AD.

These divergent metabolic requirements of Tregs and microglia may create distinct, context-dependent vulnerabilities to metabolic modulation in AD pathogenesis. Recent preclinical studies in 5xFAD mice demonstrate that rapamycin reduces CD11c+ microglial activation yet increases amyloid plaque load (Ouk et al., 2026), revealing the nuanced effects of mTOR inhibition. Similarly, pilot clinical data show transient increases in CSF inflammatory markers in AD patients (Gonzales et al., 2025), highlighting the difficulty in translating preclinical findings.

Mitochondrial integrity is equally critical for Treg suppressive function. Studies in systemic autoimmune contexts demonstrate that mitochondrial oxidative stress compromises Treg stability through DNA damage responses and mitophagy defects (Alissafi et al., 2020). In AD, microglial NLRP3 activation, which may be driven by tau species and potentially by oxidized mitochondrial DNA, could contribute to enhanced tau phosphorylation through IL-1β signaling (Ising et al., 2019).

A critical unresolved question is whether mitochondrial dysfunction in CNS-infiltrating Tregs similarly compromises their suppressive function in AD, and whether this impairment exacerbates microglial NLRP3-mediated neuroinflammation through loss of Treg-mediated metabolic or cytokine-mediated immunosuppression. This intercellular metabolic crosstalk represents a promising avenue for understanding AD pathogenesis.

### 4.5 Integrative perspective: stage-dependent dynamic evolution of Treg–microglia interactions

The crosstalk between Tregs and microglia in AD is not static but undergoes profound stage-dependent remodeling that parallels disease progression. Integrating the molecular mechanisms discussed above, a dynamic framework emerges in which the balance between neuroprotection and neuroinflammation shifts as pathological burdens accumulate, transitioning from coordinated immune surveillance to mutual cellular exhaustion.

In the early stage of AD, when Aβ oligomers initiate deposition and tau pathology remains limited, microglia retain a homeostatic surveillance phenotype with preserved phagocytic capacity mediated by the TREM2-APOE signaling axis (Zhou et al., 2020). Functional Tregs infiltrate the CNS parenchyma via the choroid plexus pathways (Baruch et al., 2015), where they accumulate at sites of Aβ plaque pathology and express IL-10 (Dansokho et al., 2016). IL-10 signaling via the JAK1-STAT3 pathway suppresses NLRP3 inflammasome priming and limits the release of IL-1β and TNF-α, thereby preventing collateral neuronal damage while maintaining microglial phagocytic competence (Kim and Lee, 2026). Concurrently, TGF-β signaling (predominantly via SMAD4/SALL1 axis) promotes the homeostatic signature of microglia (Li et al., 2024). Contact-dependent mechanisms involving CTLA-4–mediated trogocytosis of CD80/CD86 on APCs [including microglia as extrapolated from DC/B cell data] and subsequent modulation of PD-1/PD-L1 pathways further dampen neuroinflammatory signaling (Tekguc et al., 2021). At this stage, the Treg–microglia axis functions as a protective feedback loop, mitigating initial neuroinflammatory insults and preserving cognitive function (Baek et al., 2016).

As AD progresses to the intermediate stage, the escalating burden of Aβ plaques and emerging tau pathology create a chronically inflammatory CNS milieu that progressively erodes Treg function. While peripheral Treg percentages remain stable, the suppressive capacity of Tregs is significantly compromised in AD patients, accompanied by reduced CD25 expression and trends toward diminished IL-10 secretion (Faridar et al., 2020). Concurrently, microglia undergo a TREM2-dependent transcriptional transition toward the DAM and MGnD states, characterized by upregulation of phagocytic machinery (e.g., APOE, Cst7) and loss of homeostatic markers (e.g., P2ry12, Tmem119); however, this phenotypic switch represents a dysfunctional adaptation where microglia lose tolerogenic functions rather than simply acquiring pro-inflammatory effector functions (Krasemann et al., 2017; Ulland and Colonna, 2018). This phenotypic shift is accompanied by metabolic reprogramming from oxidative phosphorylation to aerobic glycolysis, driven by mTOR-HIF-1α pathway activation and mitochondrial dysfunction, which compromises lysosomal acidification and Aβ clearance efficiency (Baik et al., 2019; Marschallinger et al., 2020). Treg-mediated immunosuppression is compromised in Alzheimer’s disease, impairing the suppression of pro-inflammatory cytokines such as IL-6 (Faridar et al., 2020) Concurrently, sustained NLRP3 inflammasome activation drives caspase-1-dependent IL-1β maturation in AD pathology (Heneka et al., 2013). Notably, the metabolic insufficiency of exhausted Tregs—characterized by mitochondrial oxidative stress and defective mitophagy—further exacerbates the pro-inflammatory microenvironment, creating a bidirectional feedforward loop of immune dysregulation (Alissafi et al., 2020).

**FIGURE 1:**
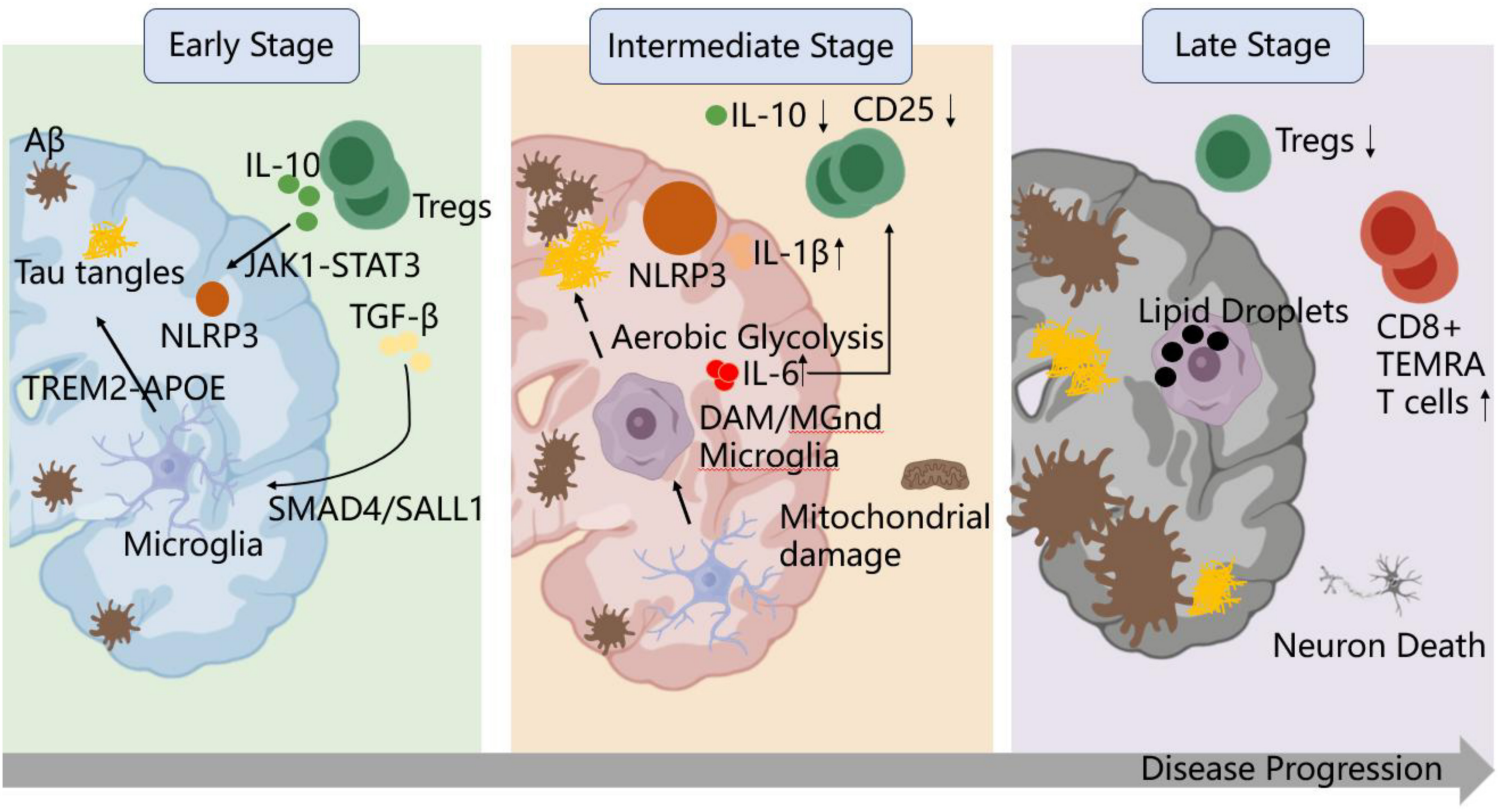
Temporal dynamics of Treg–microglia crosstalk across AD stages. The interaction transitions from coordinated neuroprotection (early), through metabolic and functional compromise (intermediate), to mutual exhaustion and severe immunodysregulation (late).

In the late stage of AD, both Tregs and microglia exhibit profound dysfunction and numerical depletion. Treg immunomodulatory mechanisms are compromised in Alzheimer’s disease, with significantly reduced suppressive capacity and decreased CD25 expression essential for maintaining Treg functional homeostasis (Faridar et al., 2020, 2022). Microglia accumulate lipid droplets and transition into a dysfunctional, pro-inflammatory state with aberrant lipid metabolism (Marschallinger et al., 2020). Disruption of the blood–brain barrier permits clonally expanded, antigen-experienced cytotoxic CD8^+^ T effector memory CD45RA+ (TEMRA) cells to infiltrate the cerebrospinal fluid and perivascular brain regions, where their cytotoxic effector functions—including granzyme release and pro-inflammatory cytokine signaling—amplify microglial activation and perpetuate neuroinflammation in AD (Sweeney et al., 2018; Gate et al., 2020). See Figure 1 for the schematic diagram of temporal dynamics of Treg-microglia crosstalk across AD stages.

Paradoxically, while transient Treg depletion in 5xFAD mice has been associated with enhanced Aβ clearance and improved cognition by lifting systemic immunosuppression (Baruch et al., 2015), other studies indicate that Treg depletion—particularly at early disease stages—accelerates cognitive decline, highlighting the dichotomous and stage-dependent effects of Tregs in modulating neuroinflammation (Baek et al., 2016). This late-stage scenario underscores the context-dependent and biphasic nature of Treg function: immunosuppression that is protective early in disease progression becomes detrimental when phagocytic clearance mechanisms are critically needed, yet the cellular exhaustion that characterizes advanced disease precludes simple therapeutic restoration (Dansokho et al., 2016; Jie et al., 2025).

This stage-dependent model reconciles seemingly contradictory experimental findings and highlights the necessity for temporally precise therapeutic interventions. Early stage strategies should aim to preserve or enhance Treg function and IL-10/TGF-β signaling to sustain microglial phagocytosis and dampen initial neuroinflammatory insults. In contrast, intermediate to late-stage approaches may require metabolic modulation—targeting mTOR activity or restoring mitochondrial integrity—to rejuvenate exhausted microglial states independent of Treg-mediated suppression. Understanding this dynamic evolution is essential for translating Treg–microglia crosstalk into clinically effective, stage-appropriate immunotherapies for AD. However, the stage-dependent mechanistic framework outlined above is largely constructed from transgenic rodent models with rapid amyloid deposition, and the temporal dynamics, microglial transcriptomic responses, and Treg stability under chronic inflammation may differ substantially in the slowly progressive, aging-associated context of human sporadic AD.

## 5 Therapeutic potential of Tregs–microglia crosstalk in AD

The dynamic crosstalk between Tregs and microglia offers immunoregulatory targets with significant translational potential for AD treatment (Dansokho et al., 2016). The regulatory network, which consists of cytokine-mediated signaling, surface molecule interactions, and reciprocal phenotypic modulation, plays an important role in maintaining neuroinflammatory homeostasis. Strengthening their protective crosstalk has become a central direction for AD immunotherapy (Yang et al., 2022).

In APP/PS1 models, Tregs depletion is associated with decreased microglial accumulation around Aβ plaques and exacerbated early cognitive impairment, whereas low-dose IL-2-mediated Tregs expansion can partially reverse these effects (Dansokho et al., 2016). Adoptive transfer of Aβ-specific Tregs effectively inhibits abnormal microglial activation, reduces Aβ plaque accumulation and tau phosphorylation, further verifying the core value of their crosstalk in AD treatment (Yang et al., 2022). It is important to note that these findings were obtained in transgenic mouse models that recapitulate only limited aspects of human AD pathology. Key differences between murine and human immune systems—including Treg phenotypes, microglial heterogeneity, and disease progression timelines—necessitate caution when extrapolating these results to clinical applications.

Building on preclinical findings, low-dose IL-2 therapy has advanced into clinical investigation. A Phase 1 pilot study (*n* = 8) established safety and feasibility, showing that subcutaneous IL-2 expanded peripheral Tregs and reduced circulating inflammatory mediators (Faridar et al., 2023). A subsequent Phase 2a trial (*n* = 38) revealed that intermittent dosing (every 4 weeks) outperformed more frequent administration by avoiding Treg exhaustion, achieving sustained Treg expansion, greater suppression of inflammatory chemokines (CCL2, CCL11), elevated CSF Aβ42 levels (potentially indicative of altered amyloid dynamics, though the precise interpretation requires further investigation), stabilized neurofilament light chain, and a trend toward slower cognitive decline (Faridar et al., 2025). However, these trials primarily assessed peripheral immune parameters; the direct effects of IL-2-expanded Tregs on microglial phenotypes in the human brain remain to be elucidated. Given the small sample sizes, these results represent a promising but preliminary therapeutic approach. Several ongoing trials (NCT05468073, NCT06096090) are further evaluating these interventions (Jie et al., 2025). Beyond systemic administration, biomaterial-mediated delivery systems such as polyethylene glycol hydrogel-encapsulated Tregs may improve cellular enrichment at brain lesions while minimizing systemic side effects (Santos Roballo et al., 2019), though their translation to human therapy faces additional challenges including blood-brain barrier delivery and long-term safety validation.

The Tregs-microglia interaction also extends beyond classical immunosuppression. Mechanisms extrapolated from non-AD CNS injury models suggest that Osteopontin secreted by Tregs binds integrin receptors on microglia, enhancing repair activity and promoting oligodendrogenesis. Furthermore, amphiregulin (AREG), highly expressed by brain-resident Tregs, blocks the microglial IL-6/STAT3 pro-inflammatory pathway through EGFR signaling (Ito et al., 2019). These non-immune mechanisms have been primarily characterized in rodent models or *in vitro* systems, and whether human brain-resident Tregs exhibit similar functional capacities remains under active investigation.

The literature also presents a “Treg Paradox”: while some studies suggest boosting Tregs alleviates neuroinflammation (Alves et al., 2017; Yang et al., 2022), others argue that transient FOXP3^+^ cell depletion may enhance Aβ clearance by allowing peripheral immune recruitment (Baruch et al., 2015). This discrepancy likely arises from different disease stages and mouse strains. Treg enhancement may benefit the neuroinflammatory peak, but excessive early immunosuppression could hinder innate clearance mechanisms—a nuance often overlooked in preclinical protocols. Resolving this paradox requires defining stage-specific intervention windows, employing temporally controlled Treg manipulation, and developing biomarkers to identify optimal patient populations. The clinical finding that intermittent Treg stimulation outperforms continuous dosing may offer one approach to balance these competing needs (Faridar et al., 2025). Nonetheless, these therapeutic strategies were predominantly validated in young transgenic mice, and their translation to elderly human patients—whose Treg homeostasis is compromised by immunosenescence and whose microglial responses diverge from murine counterparts—requires careful validation in humanized systems before clinical application.

## 6 Critical assessment of current model systems and translational relevance to human AD

While extensive preclinical studies have provided a foundational understanding of the crosstalk between regulatory Tregs and microglia, a critical assessment reveals significant limitations in translating these findings to the human condition. The vast majority of data are derived from transgenic rodent models (e.g., APP/PS1, 5xFAD) (Drummond and Wisniewski, 2017). These models predominantly simulate early onset familial AD driven by amyloid precursor protein over-expression, fundamentally failing to recapitulate the complex, multi-factorial pathogenesis of sporadic AD, which accounts for over 95% of human cases. Furthermore, human aging—a primary risk factor for sporadic AD—is accompanied by profound immunosenescence. Rodent models, often evaluated at relatively young chronological ages, lack the age-related exhaustion and functional decline of both peripheral Tregs and central microglia observed in elderly human patients.

A major barrier to clinical translation lies in the distinct transcriptomic and functional divergence between murine and human immune cells. Recent single-cell RNA sequencing studies have demonstrated that the disease-associated microglia signatures in mice only partially overlap with human microglial responses in AD brains (Zhou et al., 2020). Similarly, the immune cell composition and differentiation states in specific-pathogen-free laboratory mice differ significantly from those in adult humans, resembling neonatal immune profiles (Beura et al., 2016). Therapeutic strategies that effectively modulate Treg-microglia crosstalk in rodents, such as low-dose IL-2 expansion or ex vivo Treg adoptive transfer, retain substantial efficacy in preclinical human cell–mouse chimeric models, where ex vivo expanded human Tregs robustly suppress microglial activation and alleviate amyloid burden, despite potential constraints arising from murine MHC restriction and xenogeneic cell specificity (Faridar et al., 2022). The data generated from these animal models, therefore, must be interpreted with caution, as they may oversimplify the spatial-temporal dynamics of the neuroimmune microenvironment in human AD.

To bridge this translational gap, the field must urgently pivot toward more sophisticated humanized model systems. Induced pluripotent stem cell (iPSC)-derived 2D co-cultures and 3D cerebral organoids offer promising avenues to study patient-specific microglial phenotypes (Abud et al., 2017). These advanced platforms further enable investigations into interactions between microglia and human Tregs (Shan et al., 2026). However, a critical limitation of current brain organoid systems is the intrinsic absence of a functional vascular network and peripheral immune infiltration, which restricts their utility in modeling neuroimmune interactions (Song et al., 2026). Future research should prioritize the development of vascularized organoids on a chip or humanized chimeric mouse models (engrafted with human immune and glial cells). Only by rigorously validating findings in these advanced, human-relevant systems can we determine whether targeting Treg-microglia crosstalk represents a viable disease-modifying strategy or merely an artifact of murine biology (Table 2).

**TABLE 2:** Comparison of experimental models in Treg-microglia research.

Model system	Addressable scientific questions	Critical limitations unaddressable	Key advantages	Translational relevance
Transgenic mice (APP/PS1, 5xFAD)	• Stage-specific effects of Treg depletion/expansion on amyloid pathology (e.g., the paradox of early-stage neuroprotection vs. late-stage clearance enhancement) • Dynamic interplay between systemic immunosuppression and CNS neuroinflammation • Association between peripheral Treg functional states and cognitive phenotypes	• Sporadic AD pathogenesis: Cannot recapitulate aging-associated, multifactorial etiology with metabolic comorbidities • Human immunosenescence: Lacks context of Treg functional exhaustion and epigenetic instability under chronic inflammation • Transcriptomic divergence: Murine DAM/MGnD signatures only partially overlap with human microglial responses • Disease kinetics: Models aggressive early onset amyloidosis rather than decades-long progression of human sporadic AD	• Rapid plaque formation enabling longitudinal intervention studies • Clear genetic background supporting conditional knockouts and fate mapping • Intact blood-brain barrier and neurovascular unit architecture	Low to moderate: mimics familial early onset AD only; murine Tregs exhibit greater stability than human Tregs under specific-pathogen-free conditions, failing to replicate human Treg dysfunction in chronic inflammation
iPSC-derived models (2D co-cultures & 3D cerebral organoids)	• Human-specific phenotypes: HLA-DR expression, human microglial transcriptomic signatures • Direct contact mechanism: Molecular interactions between Tregs and microglia (e.g., IL-10, TGF-β signaling validation)Patient-specific studies: Isogenic controls using cells from AD patients	• Absence of vascularization: Cannot model Treg transmigration across BBB or parenchymal infiltration • No peripheral immune compartment: Unable to study Treg recruitment dynamics or systemic immune-brain communication • Aging deficit: Cannot replicate age-associated immune dysfunction (inflammaging) • Maturation limitations: Microglia in 3D organoids may display immature transcriptional states	• Human genetic background supporting patient-specific mechanistic studies • 3D organoids possess human cellular complexity • Amenable to high-throughput drug screening and *in vitro* mechanistic validation	Low (2D) to High (3D): Captures human microglial molecular phenotypes but lacks systemic immune context; unable to assess Treg *in vivo* stability, homing capacity, or chronic aging effects
Humanized chimeric mice	• *In vivo* human cell function: Survival, expansion, and immunomodulatory function of human Tregs *in vivo* • Cross-species validation: Functional interactions between human immune cells and (murine or humanized) glial cells • Therapeutic Treg assessment: Preliminary *in vivo* evaluation of human Treg cellular therapy	• GvHD confounds: Risk of graft-versus-host disease and complex ethical approval processes • Cost constraints: Prohibitively high costs limiting large-scale, long-term longitudinal mechanistic studies • Engraftment efficiency: Limited efficiency and regional specificity of human microglial engraftment in murine brains	• Only *in vivo* model with functional human immune cells • Enables assessment of peripheral-CNS immune axis integrity • Allows detection of human-specific therapeutic Treg behavior *in vivo*	High (theoretical): Currently limited by technical complexity, GvHD complications, and prohibitive costs; primarily used for proof-of-concept validation rather than routine mechanistic studies

A critical translational concern that warrants explicit discussion is the risk of generalized immunosuppression in elderly AD patients, particularly those with metabolic and cardiovascular comorbidities. Aging itself is characterized by immunosenescence and chronic low-grade inflammation (inflammaging), rendering older adults more susceptible to infections and impaired vaccine responses (Franceschi et al., 2018; Frasca and Blomberg, 2020). Systemic Treg-enhancing interventions—such as high-dose IL-2 therapy or adoptive Treg transfer—may inadvertently exacerbate these vulnerabilities by further dampening anti-tumor immunity and pathogen surveillance. Moreover, comorbid conditions such as type 2 diabetes and atherosclerosis are themselves associated with dysregulated immune-metabolic homeostasis (Franceschi et al., 2018); broad immunosuppression in this context could theoretically worsen glycemic control, increase infection risk, and accelerate vascular pathology. Therefore, future clinical trials must incorporate rigorous immune monitoring, employ CNS-targeted delivery systems to minimize systemic exposure, and stratify patients based on baseline immune competence and comorbidity burden. Only through such precision-based approaches can the therapeutic window of Treg modulation be safely navigated in the vulnerable elderly AD population.

## 7 Conclusion

This review has systematically examined the bidirectional crosstalk between Tregs and microglia as a unified neuroimmune regulatory axis in AD pathogenesis. We have delineated how this interaction dynamically evolves across disease stages—from an early “protective” mode characterized by Treg-mediated IL-10/TGF-β signaling that sustains microglial phagocytic capacity, through an intermediate “imbalanced” phase marked by declining Treg function and pro-inflammatory microglial polarization, to a late “dysregulated” state in which the exhaustion of both cell populations drives irreversible neuronal loss. This stage-dependent framework reconciles seemingly contradictory findings in the literature and underscores the necessity for temporally precise immunomodulatory interventions rather than uniform therapeutic approaches.

Moving forward, the most critical challenge remains the translational gap between murine preclinical data and the human condition. As emphasized throughout this review, the striking divergences in microglial transcriptomic signatures, Treg epigenetic stability, and the pervasive influence of human inflammaging severely limit the direct extrapolation of rodent findings to clinical settings. The field may benefit from transitioning toward multi-platform validation strategies—integrating single-cell and spatial multi-omics analyses from post-mortem human AD tissues with emerging iPSC-derived organoid and humanized chimeric mouse systems—to capture human-specific neuroimmune dynamics more faithfully. Furthermore, future therapeutic strategies targeting the Treg-microglia axis, such as low-dose IL-2 or adoptive Treg transfer, should not be treated as a “one-size-fits-all” approach; precision medicine frameworks that account for patient age, metabolic comorbidities, disease stage, and individual neuroinflammatory profiles will likely be essential. Only through this rigorous, human-centric paradigm can the intricate Treg-microglia crosstalk be responsibly harnessed to develop stage-appropriate, disease-modifying immunotherapies for AD.
